# Effects of a community health promotion program on social factors in a vulnerable older adult population residing in social housing

**DOI:** 10.1186/s12877-018-0764-9

**Published:** 2018-04-16

**Authors:** Gina Agarwal, Madison Brydges

**Affiliations:** 1 Departments of Family Medicine, Clinical Epidemiology and Biostatistics, Family Medicine Residency Program, McMaster University, David Braley Health Sciences Centre, 100 Main Street West, 5th Floor, Hamilton, ON L8P 1H6 Canada; 2 Department of Health, Aging and Society, McMaster University, 1200 Main St W, Hamilton, ON L8S, 4L8 Canada; 3 Department of Health Research Methods, Evidence and Impact, McMaster University, 1200 Main St W, Hamilton, ON L8S, 4L8 Canada

**Keywords:** Health promotion, Older adults, Social support, Social connectedness

## Abstract

**Background:**

Supporting older adults’ health and wellbeing in the community is an important policy goal that can be supported by health promotion. Despite widespread acceptance of the biopsychosocial model of health and its relation to health, many health promotion programs fail to realize this model in program design. Further, there is limited evidence to support program design targeting social determinants of health such as social isolation or connectedness. To fill this gap, we aimed to understand older adult’s experiences participating in cardiovascular health promotion program in a subsidized residential building to capture unintended ‘spin-off’ psychosocial effects.

**Methods:**

This study took a constructivist, ethnographic approach utilizing participant observation and semi-structured interviews with participants of the program to understand participant’s lived experiences of a health promotion program. In total, we conducted eighty hours of field work and fifteen semi-structured interviews with participants of the program. Thematic analysis was used to analyze the data.

**Results:**

Four themes emerged. First, the health promotion program filled a perceived gap caused by a constrained and impersonal health care system. Secondly, the program connected older adults with resources and provided regular and secure access to health information and support. Third, for some residents, the program facilitated social relationships between older adults, leaving participants feeling more socially connected to other residents. Lastly, a paradox of loneliness emerged where older adults talked openly about feelings of loneliness, however not in relation to themselves, but rather regarding their peers.

**Conclusions:**

Psychosocial aspects of health, such as loneliness, social connectedness, and social support may be of equal value as the physical health benefits to the older adults who participate in health promotion programs. Incorporating these elements into programming is a complex goal, and the complexity of targeting social determinants of health such as social loneliness or connectedness should not be under-estimated. Given the benefits of targeting social determinants of health, future research should be considered that measure both the objective and subjective aspects of social isolation, loneliness and connectedness in health promotion programming.

## Background

Improving the health and wellbeing of community-dwelling older adults continues to be a social policy priority [1–3]. In addition to physiological health, numerous other factors impact individuals’ wellbeing as they as age, including socioeconomic status, socio-demographic, and socio-psychological factors, such as social network size, social support, and being socially isolated [4, 5]. There is strong evidence that being socially supported and connected has implications for older adults’ health and wellbeing and quality of life as they age [6–9]. Social isolation is an objective measure of the amount of contact an individual has with their social networks [10, 11], while loneliness refers to the subjective feeling of emotional isolation caused by an absence of meaningful and sustained communication [7].

The biopsychosocial model of health implies that health promotion programs should target both biomedical and psychosocial factors related to health and wellbeing [12]. However, there is both weak and limited evidence of the efficacy of health promotion programs in targeting specific social problems amongst older adults, such as social isolation, loneliness, and social participation. Several systematic reviews of interventions targeting social isolation have concluded that many of these studies are restricted by poor methodological design [11, 13, 14]. However, there is evidence that several factors result in favourable outcomes, such as including group interventions over individual activities, and having knowledgeable and trained individuals delivering the intervention [14]. Raymond et al., who reviewed interventions targeting the social participation of seniors, suggest that interventions should be located in close proximity to the target population, that interests, culture, and beliefs of older adults be incorporated into the program scheme, and that interventions aim to support the meaningful development of older adults social relationships and roles [8].

Despite widespread acceptance of the biopsychosocial model of health, few health promotion programs have adopted such a framework in program design, implementation, or outcomes [15]. In response to this gap, this research adopted multi-dimensional methodology that included a randomized control trial [16] and ethnographic methodology to gain a more holistic understanding of one particular health promotion program. Using these methods, this research aimed to identify several indirect impacts of a cardiovascular health promotion program targeting older adults, such as individual perceptions of loneliness, social participation, and other social or individual level factors.

### Study setting

The Community Health Assessment Program by Emergency Medical Services (CHAP-EMS, subsequently renamed CP@clinic or 'Community Paramedicine at Clinic'), developed by McMaster University in Hamilton, Ontario, Canada,^1^ [16], is a health promotion program that is delivered weekly by community paramedics to residents living in subsidized older adults’ housing buildings. The residential building where the CHAP-EMS/CP@clinic program was held had 260 apartments and 84.5% of residents in the building were over 65 years of age. The majority of the residents were classified as low income, meaning their rent was subsidized by the local municipality. The building was chosen to receive the program due to large emergency (911) call volumes to Emergency Medical Services (EMS). More detail on the program characteristics can be found in two previous publications [16, 17].

At the time of this study the program had been operating for approximately two years. Residents voluntarily enrolled in the program and attended the sessions weekly to have cardiovascular, metabolic and fall risks assessed, and tailored lifestyle education delivered. The program also connected older adults with local health and community resources such as home care services and sent their risk assessment information to their primary care physician, with consent. The original goal of the CHAP-EMS/CP@clinic program was to improve individuals’ cardiovascular health through blood pressure and diabetes risk monitoring and reduce emergency department usage measured by EMS call volumes [16].

The CHAP-EMS/CP@clinic program was held in a common area of the residential building, which had a large space with couches and a porch, and a private area where one-on-one sessions with the paramedics were held. The program was informal in that residents did not need to make an appointment to see the paramedics and the time they spent one-on-one with the paramedics was not constrained or limited. At any point during the day that the program was operating (an eight hour time period) residents would wait in the common area until it was their turn to be seen in a private area with the paramedics. As there were approximately seventy individuals enrolled in the program, at times there were five to ten individuals waiting in the common area.

During the one-on-one time with the paramedics, the participants would have their blood pressure measured and their health and wellbeing and any ongoing concerns discussed. While there was a protocol for the paramedics to follow in order to record the residents’ blood pressure into a standardized format, the residents or paramedics also had the time to discuss the residents health. These conversations were private and not held in the presence of the other residents waiting to be seen by the paramedics or the researcher.

At the time of this study, two to three paramedics were present each week operating the CP@clinic sessions. The paramedics operating the program were “modified” paramedics (paramedics who for a variety of reasons, including having an acute or chronic injury, were not working as a paramedic on an ambulance). Several of the paramedics had operated the program for over a year. The program is still in operation at the date of this study, though it has been permanently re-named as CP@clinic.

### Aims

The original study of the CHAP-EMS/CP@clinic program aimed to decrease EMS calls and improve cardiovascular health and lifestyle [16]. However, to gain a holistic view of the program and capture the program’s ‘spin-off’ effects, this research took an ethnographic methodological approach. Ethnography is a widely-used research methodology that involves the researcher becoming embedded in a social setting - primarily through participant observation - in order to develop an in-depth understanding of a social phenomenon [18]. Although ethnography has its roots in anthropology and sociology, the methodology has become increasingly common in health care research. In the study of health care, ethnographic research can provide an in-depth understanding of how social processes impact the delivery and experience of health care [18]. Consistent with these goals, this study was situated within a constructivist paradigm and as such, aimed to provide a rich description and understanding of the CHAP-EMS/CP@clinic program from the perspective of the individuals’ attending it [19]. A constructivist approach was an ideal epistemological framework for understanding how individuals construct meaning from their daily lived experiences and interactions with others.

The goal of this paper was to distill the individual and group impacts of the program in order to arrive at a holistic understanding of health promotion program functioning in this vulnerable seniors’ population.

## Methods

This research took the form of an ethnography, the details of which have been thoroughly described in a previous publication [17]. Consistent with an ethnographic research design, data collection included ethnographic participant observation complemented by semi-structured interviews to understand residents’ experiences and perceptions of the program.

From March to June 2014, ten CHAP-EMS/CP@clinic sessions were attended, resulting in 80 h of field work. As the main goal of the research was to understand individual’s experiences of attending the program, individuals were recruited to participate in semi-structured interviews based on their attendance at the community health sessions with the goal of including both individuals who attended the sessions weekly and those who attended more sporadically. The community paramedics who operated the program aided in identifying individuals who were regular or inconsistent users of the program. Demographic information, such as age, gender, and health status was used to ensure a wide variety of participants were included. Throughout this period, fifteen individuals were recruited and took part in semi-structured interviews. All of the individuals approached agreed to take part in an interview. Interviews were audio-taped and transcribed verbatim before analysis.

As this study was exploratory, the initial three weeks of participant observation were unstructured to allow for time to observe and decide which social phenomenon was of greater interest [20]. Following the initial study period, observation of group social interactions between residents and the community paramedics was conducted in the common room adjacent to the program location, as this was where the majority of interactions between residents and community paramedics were had (versus one-on-one interactions with the community paramedics held in the private area reserved for the program).

On the first day of participant observation the residents were introduced to the researcher (MB) by the paramedics and oriented to her role. Residents were already aware of the intent to evaluate the program. They were informed that in this portion of the study, the researchers were interested in participants’ experiences of using the program and its impacts outside of their individual health (i.e. social interactions with their neighbours). Each week when new residents attended, the researcher would introduce herself and her role and address any questions about the research.

During the sessions, the researcher would sit in the common area with the residents as they waited to be seen by the paramedics. She would enter into conversation with the residents and the paramedics in group and one-on-one discussions. There were several social events that were had during the participant observation sessions (i.e. a “show and tell” from a paramedic who used to staff the program) that the researcher attended and participated in. Short notes were taken throughout each session where appropriate, and detailed field notes were transcribed immediately following each session.

Inductive thematic analysis was used by the researchers (GA, MB) to analyze the semi-structured interviews and field notes. Inductive thematic analysis involves an iterative approach where the researcher conducts data collection and analyses simultaneously [21, 22]. Transcripts were separately analyzed by both researchers (GA and MB) and themes were generated based on joint consensus. This approach allowed for the incorporation of new themes and pursuing new lines of questioning as they emerged, with continual reference back to the original transcripts and notes.

## Results

In total, nine females and six males were interviewed (18% of the total number of residents who attended the program), with ages ranging from 63 to 89. Three of the participants lived with a spouse and the remainder were single or widowed. All participants interviewed had at least one health problem. Twelve out of the fifteen participants had hypertension and several had an additional comorbid condition, such as diabetes or chronic obstructive lung disease. Thirteen participants had been using the program for over two years, however all participants varied in their weekly attendance. Our interview sample (60% female, 30% male) was similar to the overall gender composition of the residents attending the program (68% female, 32% male). At the time of this study there were 79 residents in the CHAP-EMS/CP@clinic program (34.8% of the total residents in the building). Of these, 90% of participants had a family doctor, and the majority were of low socio-economic status.

This research identified four dominant themes that can be described as direct and indirect health effects. The direct effects were: the CHAP-EMS/CP@clinic program filled a health care need; and the program facilitated access to health knowledge and resources. The indirect health effects were: the program brought changes, and challenges to improving social connectedness amongst participants; and older adults expressed varying degrees of loneliness amongst themselves and their peers. While the first two themes address how the program directly impacted individuals health, the third and fourth themes discuss the social aspects of the program as an indirect means of achieving or improving health.

### 1. Filling a health care ‘need’

As participants spoke of their health care experiences, it was clear that many perceived the CHAP-EMS/CP@clinic program as filling a ‘need’ in their current access to health care. For many participants, their experiences within the CHAP-EMS/CP@clinic program contrasted sharply with their interactions with other elements of the health care system or their health care provider. As such, the program provided a way to mitigate their frustrations with the health care system, which were mainly concerned with a lack of time with their primary medical provider. Participants often stated that during the program, paramedics took time to address their concerns and they felt they could ask for help about any problems they had. Their interactions were personal and they felt that they were being listened to. Further, because the program operated on a weekly basis, they saw the CHAP-EMS/CP@clinic paramedics more frequently than other health care providers.

Participants expressed views that there was simply not enough time in the current health care system to address all of their questions or concerns, hence they had unmet ‘needs’. They often discussed situations in which they felt their health concerns had not been taken seriously, misdiagnosed, or missed entirely. At times, they had difficulty making an appointment or seeing their family doctor as often as they would have liked and felt they had to find other avenues to address their health care needs. Further, participants expressed concerns at a broader level, expressing that their frustrations with the overall health care system itself often overshadowed positive interactions with their family doctor. Participant 109 stated:

*“I mean when you have your ten minute doctor’s appointment you get one question and that’s it. I mean he is a great doctor. But they don’t have time. Unfortunately the system doesn’t have time.”*

In contrast to these concerns and experiences, participants who attended the CHAP-EMS/CP@clinic program stated they valued the ability to have all of their questions and concerns discussed at once, at a time that was convenient for them. Gradually, participants developed relationships with the paramedics to the point that they stated they trusted them. Previously hidden social, medical or mental health problems emerged, and support was provided. The one-on-one relationships between the residents and paramedics were described as close and residents felt they were “being taken care of”. We frequently heard these sentiments voiced:

*Participant 101: “I am sure he is a good doctor, but he doesn’t have the people skills that I need. He was an emergency room doctor and I don’t think he realizes he is actually going to see us again. We aren’t one offs you know (laughter). So unless you are in crisis he isn’t really aware of what is going on. He is blasé about it. The last three or four times I have gone in the diagnosis I have got is that I am “old”. I don’t process that as a diagnosis. So what do I do? I just use the other systems.”*

Some residents had started to use the program as an alternative to seeking medical attention in other settings, such as the hospital or their family doctor. Many of the residents who were reluctant to go to the hospital for medical concerns trusted the paramedics to advise them on the best course of action for their health. The program offered flexibility and options when making decisions about their health, and a more convenient and pleasant alternative to seeking care elsewhere. For some residents, if they were not feeling well throughout the week they would wait until the next CHAP-EMS/CP@clinic session to address the problem. Participant 107 stated:

*“A lot of people don’t wish to go to the doctor. And I thought well this is a good way for them to be monitored without actually having to go to their doctor.”*

For some participants the CHAP-EMS/CP@clinic sessions were readily embraced as part of how they managed and maintained their health, instead of relying on their family doctors, the emergency department, or other health care providers. While none of the participants reported that they no longer valued physician services, the CHAP-EMS/CP@clinic program was able to provide an additional health care service that addressed unmet needs such as having a long appointment and receiving additional support, that participants perceived were unavailable in the current health care system. In circumstances where the paramedics had not been able to deal with the medical issues, they had facilitated the patient’s next appointment with the family doctor, or urgent transport to the emergency department as appropriate. Residents felt appreciative of this extra communication that had been made by the paramedics with the health care system.

Several program features facilitated benefits for residents: the program was available to all residents regardless of their current health status; participants had a flexible time to see the paramedics without making an appointment; they were not limited to seeing the paramedics only once during a single session; they had access to a minimum of one and often two paramedics with whom to discuss their health; and they were able to form long-term relationships with them.

### 2. Access to health knowledge and resources

Consistent with the program’s original goals, CHAP-EMS/CP@clinic provided participants with knowledge about their health and access to resources to help them learn more about their health. Those who were concerned about their current and future health could learn how to regularly and reliably monitor it. In general, participants had an interest and concern about their health. This was expressed both by those with chronic health conditions, and those who claimed to be healthy, with only minor health problems. Participant 107 stated:

*“Just the idea that people have to be aware of their blood pressure, their sugar problems and it keeps them on a weekly basis about their problem so that they keep on top of it. It’s easy to forget and just ignore it. That’s the biggest thing, catch it early, get on top of it.”*

Some felt that although they were healthy now, they wanted the program to remain in the building in case they needed it in the future. Interestingly, many of the participants denied currently requiring other community support resources, such as home care services. This suggests that for some older adults, having access to out-of-home supportive health and social services is beneficial. Participant 104 stated:

*“I am always interested in health and everything. It feels good that someone is there to help you if you have any questions because you know, I am getting older. Who knows what types of questions I might have. So that feels good.”*

Another motivator for residents to attend was the ability to seek advice or clarification on a variety of short and long term health care issues, such as chronic health problems, medication changes, and new injuries. For some, when they were uncertain about their complex medical problems they valued the advice they were given at the program. Participants 115 stated:

*“I talk to them generally about everything. Basically, I still deal with specialists because of my conditions every three months. But if anything happens to me in between then I go and talk to them and see what is going on. And they will let me know what I should do.”*

The sessions with the paramedics did result in some lifestyle changes, such as maintaining or initiating a healthier diet, changes to a daily routine, or exercise regimens. Upon following the advice of the paramedics to be more conscientious following her heart operation, one resident stated she now took the elevator instead of the stairs, in case a medical event occurred while walking.

For many participants, the health advice the paramedics gave them, such as to ask their doctor about certain diseases or concerns, made the most impact. For example, new diseases or medical problems were diagnosed, unaddressed mental health problems were discussed, and clarifications about their health problems were made. For example, Participant 110 stated:

*“They gave me some information when I had this lump on my leg. I thought it was just “part of the process”. But he [the paramedic] wanted me to go to get it checked. So I did. I wouldn’t have gone if he didn’t tell me to do it.”*

For one participant who had an undiagnosed mental health problem, the program also addressed analogous personal issues that equally impacted her life:

*Participant 111: “I was having problems with my apartment, and I was talking to him [the paramedic] about it and with that they were able to transfer me to another apartment. So yes, he helps us outside of medical reasons.”*

The program also made residents more aware of their health. This was a combination of both knowledge gained from the paramedics at the program, and being given advice from the paramedics on who to ask for more information, such as a specialist. Participant 109 stated:

*“It made me more aware of my blood pressure. We went out a bought a good blood pressure monitor so we can check. So in that sense yes, it has helped. I am curious. I want[ed] an understanding of why it [my blood pressure] goes up and down.”*

Following participation in the program, participants expressed that they were more informed and aware of their own health problems and how to manage them. While some residents admitted they were unlikely to change some elements of their lifestyle, such as diet or exercise, they still valued the CHAP-EMS/CP@clinic sessions. The program made participants feel in-control of their health and the sessions increased their personal health awareness. The feeling of being listened to, as well as having control and autonomy (rather than being at the mercy of booking an appointment) when deciding to seek medical advice, was of high value to participants.

### 3. Challenges and changes to social connectedness

The weekly CHAP-EMS/CP@clinic sessions became a place not only to receive health information and support, but to have a social gathering, organize a group event, or hear the weekly building gossip. There was an obvious change in the relationships that were formed between the building residents, highlighting the importance of the social aspect of the program in addressing older adults’ wellbeing and enhancing participation in the program.

A session usually began at 9 am with a regular group of residents waiting in the common room for the session to start. One resident often brought down coffee and home-made baked goods for the paramedics. The common area was a large space filled with couches and tables where other events were hosted in the building. The “lineup” for having blood pressure checked consisted of the residents waiting on these couches or chairs and this layout encouraged residents to socialize with each other or with the paramedics. While participants discussed their health one-on-one with the paramedics, many social interactions between residents involved discussing their health problems. Considering past conflict between some building residents, the fact that this personal information was shared openly was surprising.

Residents would also bring their family members, such as their children or grandchildren to the sessions where the small children would often play with the paramedics. A substantial portion of the regular residents who attended the sessions came down to the common room multiple times throughout the day. Participant 101 stated:

*“We all go there and see each other and say ‘how are you’ and so on. Some people you know them better and so you stick around and you talk a little bit. We all like to go when the people are here, we all go down. And some people you see in the afternoons, we go down and chat a little bit and have a good time, half an hour or what, and it gives us a break during the day.”*

While these new relationships between residents were not described as close or trusting, as those with the community paramedics, their effects should not be minimized. Following an interview with an elderly female resident who had just joined the program, another resident approached her to ask her: “Oh are you new?”. Both of the residents noted how they had been living in the building for over five years and had never met. They ended up staying in the common area following this interaction to continue socializing (field notes, April 23^rd^, 2015).

Attending the program connected residents to other events occurring in the building (such as through the building association) and also instigated new social events for residents. For example, during one session a paramedic who had previously staffed the program attended a session just to visit with the residents (field notes, June 25^th^, 2015). The visit was enjoyable for the residents who were excited to hear about her international volunteering experience that they had previously donated towards. The residents set up a food table for her visit, and even residents who were not frequent attendees of the normal CHAP-EMS/CP@clinic sessions participated in order to see her. This activity was discussed before and after the event. The contact with the paramedic had provided an extra social opportunity to look forward to, bringing residents together, not only to plan the event but to enjoy it as a group.

During the course of this study, changes to social connectedness and isolation was discussed as a benefit by all participants. Based on our observational data, it was clear that some female residents participated in the social component of the CHAP-EMS/CP@clinic program more then other residents, such as males who lived alone in the building or non-English speaking residents. This was in part because they identified as healthy and stated they did not require all of the services provided at the program at the time of the study, however enjoyed attending the program for social reasons. Several of these individuals also took on a larger role in organizing informal and formal social events in the building.

Because the program provided a vehicle for residents to discuss many of the issues that were affecting the social life and cohesion of their building, issues with the building association or other volunteer groups in the building often spilled over into the sessions. There was some negative history between building residents prior to the CHAP-EMS/CP@clinic sessions occurring resulting in hesitation for some residents to engage in social activities in the building.

Tension amongst residents and barriers to the programs’ functioning were created when the normal ebb and flow of life in the building was interrupted. There were incidents of theft in the building (in one instance there were allegations that artwork had been removed from the common area) and problems initiated by the group calling themselves the ‘Building Association’ that created conflict during CHAP-EMS/CP@clinic sessions. The Building Association, a group of residents responsible for organizing social events in the building, prior to the program being implemented, had caused turmoil between building residents. Participants stated that although things had improved since the implementation of CHAP-EMS/CP@clinic and the involvement of the paramedics assisting with the Building Organization, there remained some personal problems between residents. These problems often surfaced at the sessions and were discussed during the interviews.

Negative past experiences made it difficult for some residents to participate in other events in the building beside the CHAP-EMS/CP@clinic program. For some, this prevented them from participating in future events, even after acknowledging that many improvements to the building had been made since CHAP-EMS/CP@clinic was implemented. One participant described:

*“We play bingo every now and then. Before [another building resident] and them took it over it was just too much in-fighting. It was just terrible. They were all sniping at each other. Since they have done it, they did it right, no arguments. Here is how it is, if you don’t like it tough. So things are a lot better now. The hierarchy of the association is all changed, which was very important. Because they used to think they owned the building. It caused a lot of problems. It was a dictatorship, you couldn’t do anything.”*

The fact that the paramedics served as a mediator between residents (for example promoting conversation and actively joining in), meant that the CHAP-EMS/CP@clinic sessions remained overall a positive opportunity to meet new people and form new casual relationships. Barriers towards communication between people yielded, and there was less ‘stand offishness’ between residents, and more willingness to reach out and talk to each other, even to those with whom they had never spoken before. Participant 106 stated:

*“ It has brought people together. When I say together I mean communication. It has added a bit of social integration of people in the building. I know there are a lot of people here who don’t come out at all. But they come here. For some reason they meet and they get talking.”*

Another factor regarding whether individuals may have not participated in social events or the program in general, was language. There was also evidence from both the interviews and the observational periods that there were language barriers for some residents that may have impacted their involvement in the program. English speaking residents were aware of this barrier for other residents. One participant explained:

*“It’s the [same] people that always come down. We keep trying to encourage more people, but its basically stayed the same people. We are dealing with a language issue. Why should we leave them out? They have health problems. Look around? Do you hear any accents out there. Not many.”*

Only a three non-English speaking residents attended the program regularly. However, they had limited interactions, other than a simple greeting with other residents or the paramedics. Occasionally, English speaking family members served as translators for residents who did not speak English, however to aid the paramedics in conducting their family member’s health assessment only.

Overall, the program enhanced the social connectedness of the building by allowing residents to have an opportunity to socialize and meet other building residents, both at the sessions and during other events when the paramedics were not present. Further, residents were comfortable enough with each other to openly discuss some of their health problems. However, a history of conflict created a barriers to improving social connectedness that resulted in the relationships between residents being casual and not close, personal relationships. For a portion of residents, this history prohibited them from engaging in some types of social events entirely. While there were clear issues with regards to access and inclusivity of the social events in the building (for example, the dominant language in the building was English) residents still expressed value in forming casual relationships with others, even thought their participation in activities varied.

### 4. The paradox of loneliness

During the one-on-one interviews, participants often discussed loneliness. However, residents rarely discussed their own feelings of loneliness, but they instead discussed their peers in the building who they saw as ‘being lonely’. The common perception amongst participants was that they themselves attended the program because they enjoyed the social interactions that occurred there. However, others in the building needed the program more than they did, in particular for its social benefits, because *they* were lonely. One participant stated:

*“There is an emotion thing to say someone is there and you can go talk to them… A lot of them [other residents in the building] especially in the winter, can be too isolated.”*

Instead of discussing their own loneliness, many participants admitted to enjoying the social aspects of the program. For some, it was their primary reason for attending. This was exemplified during the observation sessions. Many participants attended the program multiple times throughout the day, not to get their blood pressure checked, but to talk with other residents and the paramedics. In this way, it is possible that isolation and loneliness felt by individuals were mitigated, since they now had something to look forward to. They would be able to interact with others, including the paramedics, in their building. One participant stated:

“*They [the other residents] look forward to a Wednesday, because now they can go and talk to somebody. That’s how bad it is here. They are lonely. But I am the opposite. I have a wife and all my grandkids and you know, I have all I need. But, I like to talk to [the paramedics]. They make themselves known.”*

Participants perceived that the program brought the building residents together in ways that had not existed before in the building. They spoke about how the program went beyond mere health and allowed other connections to happen, implying that this was of tremendous benefit to those who were lonely and isolated. They described their peers’ lifestyle as a lonely one, with little or no opportunities to go anywhere without time or monetary investment. Only three participants in our sample had a spouse and many discussed that there were limited opportunities for informal social engagement in their lives. The fact that minimal effort was required to gain this socialization was a strength of the program. There was always the possibility for those not wishing to take part that they didn’t have to. Where other activities may require more formal clothing or more preparation for residents, the idea that they could talk to someone else in their own building, without requiring a purpose or formal plans, was very restorative. Participant 112 stated:

*“I mean I am talking about our situations here. We are all on our own, we are all singles. Those people don’t get out that much or when they do, they go to the doctor, they go to shopping and that’s that. When they go out to things like the [the CHAP-EMS sessions], they don’t have to get dressed up, they just have to go downstairs and meet some people. It’s very soothing, to go down and talk to people. And I think that this should be more places like that.”*

These findings point to the underlying complexity and possible vulnerability of how older adults perceive themselves as being socially isolated or lonely.

## Discussion

The goal of this study was to capture ‘spin-off’ effects of a health promotion program in order to inform our understanding of the effect of community health programming on biomedical and psycho-social factors that effect health. This research supports the notion that health promotion initiatives can address both biomedical indicators of health and contextual level factors that influence health, such as social support, social participation, and connectedness [15, 23, 24]. Further, these may be of equal importance to the participants of the health promotion program, some of whom may be in good health but use the program for other reasons, such as a source of social support or for social interactions.

This research confirms that choice and location are important factors that contribute to participation in a health promotion program [8]. The program was located close to, and in this case, directly within, the target population’s residential building, making it convenient for residents to attend. This also allowed for participants to attend the sessions whenever they chose to, even multiple times throughout the day. The ease of access and flexibility of the program were essential strengths, particularly because they resulted in experiences that contrasted with the frustrating encounters many participants stated they had had in other areas of the health care system.

This research also contributes to the literature on social participation or relationships between older adults. Previous research has suggested that neighbourhood services and social cohesion are important for older adults’ wellbeing and can serve as a buffer to decreased social network size [25]. However, facilitating or supporting social encounters between older adults is a complex goal [26]. Research exploring this topic has found that while older adults value social interactions with their peers, they may not be concerned with having developing long-term relationships, and these relationships are easily terminated if the relationship is not reciprocated [27]. Similarly, Buijs et al. [28], found that conflict between building residents prevented some residents from attending a health promotion program. Relocation in later life, such as to a seniors’ building or assisted-living facility, may also make establishing new relationships for older adults difficult due to worsening health or a decline in physical or cognitive functioning [25, 26]. It has also been hypothesized that older adults may choose to spend their time maintaining current relationships, instead of making new ones, and are hesitant to direct too much emotional investment towards new relationships [29].

This research aligns with previous work on this topic in that study participants emphasized the importance of casual relationships with their peers, and of having an informal setting in which to carry out these activities. Interestingly, even with these features in place, participants’ physical participation in social events or attempts at forming new relationships varied considerably. Individuals recognized the difficulty in making social activities inclusive for all, and identified barriers, such as language and previous conflict between residents, that made it difficult for themselves or their peers to attend social events. However, the value of providing an environment and opportunities for enhancing social interactions should not be understated or dismissed. This research highlights that while there is variation in how older adults engage in social activities, having the option to participate, regardless of whether it is fully fulfilled, is still beneficial and can address subjective feelings of loneliness and social connectedness. This is a worthy reason to deploy a community health program in itself, in such a location.

Allowing individuals to choose the amount and type of participation in social events was a key component of the CHAP-EMS/CP@clinic program. The majority of the opportunities for social encounters between residents were informal socializing experiences. This allowed residents to choose how long they wanted to participate and was fluid enough for residents to meet others in the building they had not before. Further, the social activities that were more formal were jointly facilitated by residents in the building, rather then someone organizing the event for them. The locus of control was largely in the hands of the residents, resulting in favourable experiences for participants during this research.

The above finding was, ironically, also the source of previous conflict between building residents. However, our study also found that the presence of a mediator(s) can play an important role in managing pre-existing conflicts between residents. The paramedics operating the program took on an active role in the building and served as a buffer between residents in solving resident related disputes. This was due to the trusting and close relationships the paramedics had fostered with building residents [18], implying that the individual(s) chosen to deliver the intervention may also play an important role in facilitating its effects. Previous research has also stressed the central role of trained and knowledgeable staff in delivering health promotion programs targeting social issues [14].

Our exploratory research suggests that the CHAP-EMS/CP@clinic program was able to address social issues, such as access to social support, loneliness, while also providing access to health resources and knowledge. It is important to note that because social connectedness or loneliness were not the original goals of this program and arose organically, similar programs may not experience similar phenomena, nor may programs that directly target social connectedness or loneliness. However, by adopting an ethnographic methodology, our research highlights the importance of considering social factors, such as loneliness, support, and connectedness, as core components of health promotion programs, alongside lifestyle factors and disease prevention. Further, incorporating these components into health promotion program needs to be done in a meaningful way, paying particular attention to the historical and social context in which the programs operate within.

### Limitations

A limitation of this study is that the research team had limited interaction with and no interviews conducted with residents who did not speak English due to the lack of a translator. Although attempts were made to interact with this group, they were unsuccessful, and it is thus unknown how this demographic perceives and experiences the program. Given the large proportion of non-English speaking individuals in Hamilton (estimated to be 10.1% of all residents in the most recent census conducted in 2011 [30]), understanding how this group experiences health program programming is of interest.

Additionally, in this study, 60 % of the participants were female, were in better health, and spoke English fluently. This is not to say that this group was the only ones who benefitted from the social aspects of the program. However, this group may have experienced or used the program in ways other residents did not, such as those who had negative social experiences, were unable to communicate in English, or had poor health. Future research should also address the extent to which older adults of different age and/or health status engage in health promotion programs and their motivations for doing so (i.e. personal health or social reasons).

## Conclusions

This exploratory study sought to understand the experiences of low-income older adults residing in subsidized housing, attending a health promotion program, CHAP-EMS/CP@clinic. Through a constructivist, ethnographic methodology this research found that the program met its intended goal of connecting participants with reliable health information and support. Additionally, the program also addressed feelings of social connectedness and loneliness amongst participants, highlighting the important components of health that are often overlooked in health promotion programs: loneliness, social isolation, connectedness, and the formation of relationships between older adults. This research builds on a body of literature that [5, 6, 15] advocates for social determinants of health to be considered at the program design and implementation stages of health promotion programming. Future quantitative and qualitative research should continue to aim to understand the effectiveness of health promotion programs in addressing social issues.

## Abbreviations

CHAP-EMS: Community health awareness program through emergency medical services

## Footnotes

1 CHAP-EMS was subsequently re-named CP@clinic or Community Paramedicine at Clinic at the request of Paramedic Services in Ontario
